# Computational prediction of MicroRNAs targeting GABA receptors and experimental verification of miR-181, miR-216 and miR-203 targets in GABA-A receptor

**DOI:** 10.1186/1756-0500-5-91

**Published:** 2012-02-09

**Authors:** Chunling Zhao, Chaoqun Huang, Tingting Weng, Xiao Xiao, Hong Ma, Lin Liu

**Affiliations:** 1 The Lundberg-Kienlen Lung Biology and Toxicology Laboratory, Department of Physiological Sciences, Oklahoma State University, Stillwater, Oklahoma 74078, USA; 2 Department of Physiology, Lu Zhou Medical College, Lu Zhou, Sichuan Province 64600, People's Republic of China; 3 Department of Physiological Sciences, Oklahoma State University, 264 McElroy Hall, Stillwater, Oklahoma 74078, USA

## Abstract

**Background:**

GABA receptors are well known as the inhibitory receptors in the central nervous system and are also found in peripheral tissues. We have previously shown that GABA receptors are involved in lung development and fluid homeostasis. However, the microRNAs that regulate GABA receptors have not yet been identified.

**Results:**

In this study, we used the online software, TargetScan and miRanda, to query the microRNAs that directly target GABA receptors and then selected some of them to verify experimentally using 3'-UTR reporter assays. Computational approaches predict many microRNA binding sites on the 3'-UTR of GABA_A _receptors, but not on GABA_C _receptors. 3'-UTR reporter assays only verified miR-181, miR-216, and miR-203 as the microRNAs that target GABA receptor α1-subunit among 10 microRNAs tested.

**Conclusions:**

Our studies reinforce that microRNA target prediction needs to be verified experimentally. The identification of microRNAs that target GABA receptors provides a basis for further studies of post-transcriptional regulation of GABA receptors.

## Background

GABA receptors are well known as the inhibitory receptors in the central nervous system [1,2]. However, GABA receptors are also found in several peripheral tissues [3-6]. The functions of GABA receptors in peripheral tissues are less studied. They may be involved in ion homeostasis [7], cell proliferation and differentiation [8], development [1], and hormone secretion [5,9].

We have initially identified GABA receptor π-subunit as a specific alveolar epithelial type II cell marker through DNA microarray analysis [10]. The expression pattern of the GABA receptor π-subunit is regulated by various culture conditions and is consistent with the type II cell phenotypes [11]. We have further identified 19 subunits of the ionotropic GABA receptors in alveolar epithelial cells [6]. Their expression is dynamically changed during lung development [12]. Functionally, GABA receptors play important roles in fluid homeostasis in the adult lung and fetal lung development [6,13].

GABA receptors can be classified into two major types: GABA_A _and GABA_C _as ligand-gated Cl^- ^channels, and GABA_B _receptor as a metabotropic receptor coupled to a heterotrimeric G-protein. GABA_A _and GABA_C _receptors share a conserved structure that contains a long extracellular N-terminal region, 4 transmembrane domains (TM1-TM4), a large intracellular loop between TM3 and TM4, and a short extracellular C-terminus [1,2,14-16]. The N-terminal segment is responsible for ligand binding and subunit assembly. The TM2 domain forms the lining of the ion pore. The intracellular loop is the site for post-translational modifications and binding with other proteins. This loop harbors a number of consensus phosphorylation sites for protein kinase A and C (PKA and PKC) and tyrosine kinases [17].

MicroRNAs are small non-coding RNAs. They form a ribonucleoprotein complex, termed RISC that cleaves mRNA or represses protein translation. MicroRNAs regulate various biological processes [18,19]. Several microRNAs such as miR-17-92 cluster and miR-127 are involved in lung development [20-22]. MicoRNAs have also been implicated in many lung diseases including lung inflammation, Chronic Obstructive Pulmonary Disease, Asthma and Idiopathic Pulmonary Fibrosis [23-30]. Nevertheless, microRNAs that regulate GABA receptors have not yet been reported. In this study, we used online software, TargetScan (http://www.targetscan.org) [31] and mRanda (http://www.microrna.org) [32] to predict the microRNAs that possibly target to GABA receptors and then selected some of them to verify experimentally using 3'-UTR reporter assays. We found that miR-181, miR-23 and miR-216 target the GABA receptor α1-subunit.

## Methods

### Construction of microRNA expression vectors

Human microRNA expression vectors were constructed as previously described [22]. Mature microRNAs with the flanking sequences (~200 base pairs at each end) were PCR-amplified from human genomic DNA. The primers used for PCR amplification are listed in Table 1. The PCR products were inserted into a modified pLVX-Puro lenti-viral vector (Clontech) between CMV-driven enhanced green fluorescent protein (EGFP) and SV40 polyA terminal sequences.

**Table 1:** Primers for miRNA expression vectors

	Forward	Reverse
miR-15a	CCGCTCGAGTATTCTTTGTGTTTCCTAACCTAT	GCGGAATTCTCAATAATACTGAAAAGACTATC

miR-15b	CACCTCGAGCGGCCTGCAGAGATAATACTTC	GAGAATTCGTTGCTGTATCCCTGTCACACT

miR-16-1	GGGCTCGAGAAACATAGATTTTTTTACATGC	CCCGAATTCAATTCCTCTAATGCTGCATAAG

miR-16-2	CACCTCGAGCCTTAAAGTACTGTAGCAGCACAT	GAGAATTCGGTAAATCAAACACCAAGTGTACAG

miR-103-1	CCGCTCGAGTACTTCCCAATCCATTTAAAGTAT	GCGGAATTCAAGTAGCAGATAATTCAAATTG

miR-103-2	CGCCTCGAGTTCCAACAAATGTTTAATTACTTG	GGCGAATTCAAGTAGAGGAAGAGTGGAAGGT

miR-107	CACCTCGAGGATACAATTACAACCCATGTC	GAGAATTCCTGTCTTTCTAATATACCTCAGTG

miR-137	CACCTCGAGTTATGGATTTATGGTCCCGGTCAAG	GAGAATTCTCCAGTCCTGGTCACCAGAGGC^

miR-146a	CACCTCGAGATCCACCCACATCAGCCTTC	GGTTGAAGACTGAATTCGAGTAGCAGCAGCAGCAAGAGAG

miR-146b	CACCTCGAGTCAGCTCCAAGCTCAGACCCTC	GGTTGGTCTCGAATTCAGCACTGAGGAAGGACCAGCAT

miR-181a-2	CACCTCGAGTTCTCAGACATTCATTTGAGTC	CACGAATTCTCATCATGGACTGCTCCTTAC

miR-181b-1	GTTCTCGAGTATGACTAAAGGTACTGTTGTTTC	GCTGGTCTCTAATTTGATACTGTACGTTTGATGGAC

miR-181c	CACCTCGAGCTGCACTGCTACATCTCCATCC	GAGAATTCACACCAGCTCTCCTCTCCAAAG

miR-181d	ATTCTCGAGCTCTCCTCCTCTCCCTCTTCATGCTC	ATAGAATTCAGATTGGGCCACTGCACTCCAGC

miR-203	ATTCTCGAGGCGGGGCTGGGCTTGGCGGCTG	TTTGAATTCGCGCGCCCCCACCCAGCGGTTC

miR-216b	CCGCTCGAGTTTTCCTTCATTCTCTATGGAGAT	GGCGAATTCGATGCTAGTCTGGGAATCAAAC

miR-221	CACCTCGAGCGACCTTCCTTCCATCCAGCTT	GAGAATTCTTTGACAGTTGAGGCAGGGAGAAG

miR-424	CACCTCGAGCAGCTCCTGGAAATCAAATGGTG	GAGAATTCACTTACCCTGGCAGCGGAAACAATAC

miR-497	CACCTCGAGATGGTCCGTCGCCTTCCAGTTG	GAGAATTCTGGGTGTGGGCAACAAAGACTC

### Construction of 3'-UTR reporter vectors

The full length 3'-UTR or the microRNA binding sites in the 3'-UTR of rat GABA receptors were PCR-amplified and inserted into the pRL-TK vector containing a *Renilla *luciferase (Promega). The primers used for PCR amplification are listed in Table 2.

**Table 2:** Primers used for constructing 3'-UTR reporter vectors

Names	3'-UTR or binding Sites	Sequences
GABRA1-F-Spe I	9-1962	ggtactaGTCGTATTCTGTTGTTCAGTC
		
GABRA1-R-PspOMI		gttgggcccTCTATACATGAAATGTCCTTGG

GABRG2-F	2095-2102 (miR-103/107)	CTAGTATGGACTTTTACAATAAAAATGCTGCATTCTA
		
GABRG2-R		GGCCTAGAATGCAGCATTTTTATTGTAAAAGTCCATA

F: forward primers; R: reverse primers; microRNA binding sites are underlined

### 3'-UTR reporter assay

HEK 293T cells (2 × 10^4^/well) were seeded in each well of a 96-well plate. After one day of culture, the cells were transfected with100 ng microRNA expression vector or control vector without miRNA insert, 2.5 ng 3'-UTR *Renillia *luciferase reporter vector and 15 ng pGL3 control vector (firefly luciferase reporter) using Lipofectamine. After a 2 day transfection, the cells were lyzed and dual luciferase activities were measured using the Dual-Luciferase Reporter Assay System (Promega). The *Renilla *luciferase activities were normalized with firefly luciferase activity. Data was expressed as a ratio to the control vector without miRNA insert.

## Results and discussion

Both GABA_A _and GABA_C _receptors are ligand-gated Cl^- ^channels. However GABA_C _receptors have very unique ligand binding characteristics in comparison with GABA_A _and GABA_B _receptors, including a high sensitivity to the physiological ligand, GABA, insensitivity to bicuculline, barbiturates, and bacofen, very weak de-sensitization, a smaller single-channel conductance, and a longer open time [14-16]. Eight different subunits of GABA_A _and GABA_C _receptors (α1-6, β1-3, γ1-3, δ, θ, ε, π, and ρ1-3) have been identified. The assembly of a heteropentamer, with at least one α-, one β-, and one γ-subunit, forms functional GABA_A _receptor channels. δ-, θ-, ε-, and π-subunits can substitute for the γ-subunit. However, GABA_C _receptors are exclusively composed of ρ subunits in the form of homo- or hetero-pentamers.

To identify the microRNA that may potentially regulate GABA_A _and GABA_C _receptors, we used the online computer software, TargetScan (v 5.2) to predict the binding sites of microRNAs on the 3'UTR of rat GABA receptors. We chose rat GABA receptors because we use rats as most of our animal or cell models. Since our microRNA expression vectors use human sequences, we only queried conserved microRNA target sites among mammals based on conserved 8 mer and 7 mer sites that match the seed sequence of a microRNA. The results are listed in Table 3. Among α-subunits, we found that α1 had most of the microRNA binding sites. There were 6 binding sites for 6 microRNAs on α1 3'-UTR. For other α-subunits, we found miR-128, miR-27ab, let-7/miR-98 for α6. We did not find any microRNAs for α2, α4, and α5. There was no information available for α3.

**Table 3:** Predicted microRNAs targeting rat GABA receptor subunits by TargetScan and miRanda

GABA receptor subunits	Entrez Gene symbol	Lengths of 3'-UTR in TargetScan (v5.2)	Conserved microRNAs targeting to GABA receptors predicted by TargetScan (v5.2)	Lengths of 3'-UTR in miRanda	Conserved microRNAs targeting to GABA receptors predicted by miRanda
α1	GABRA1	2017	miR-208/208ab,	2018	miR-129 (2), miR-130b, miR-136, miR-137, miR-148b-3p,
			miR-499/499-5p, miR-181,		miR-150, miR-152, miR-181a (2) bc (3) d, miR-182,
			miR-216/216a, miR-137,		miR-186, miR-203 (2), miR-210, miR-216a, miR-26ab,
			miR-203		miR-30acde, miR-30b-5p, miR-320, miR-340-5p, miR-361,
					miR-374, miR-375, miR-376c, miR-377, miR-384-5p,
					miR-410, miR-433, miR-488 (2), miR-539, miR-874

α2	GABRA2	747	0	NA	NA

α3	GABRA3	0	0	NA	NA

α4	GABRA4	7478	0	88	miR-186, miR-200bc, miR-203, miR-429, miR-495

α5	GABRA5	586	0	880	miR-124, miR-132, miR-133ab, miR-195, miR-212, miR-223,
					miR-30acde, miR-30b-5p, miR-322, miR-346, miR-376c,
					miR-378, miR-384-5p, miR-494 (2), miR-495, miR-539

α6	GABRA6	809	miR-128, miR-27ab,	NA	N/A
			let7/miR-98		

β1	GABRB1	407	miR-30a/30a-5p/30b/30b-5p/	428	miR-103, miR-107, miR-128, miR-143, miR-148b-3p,
			30/384-5p (2), miR-103/107		miR-152, miR-30a (2) c (2) d (2) e (2), miR-30b-5p (2),
					miR-384-5p (2), miR-411

β2	GABRB2	5618	miR-203,miR-135, miR-218,	499	miR-128, miR-199a-5p, miR-203, miR-33, miR-411, miR-485
			miR-21/590-5p, miR-10,		
			miR-101, miR-19, miR-144,		
			miR-9 (2), miR-455/455-5p,		
			miR-33/33ab		

β3	GABRB3	4060	miR-26ab/1297, miR-204/211,	508	miR-122, miR-186, miR-199a-5p, miR-204, miR-210, miR-211,
			miR-23ab, miR-27ab, miR-218		miR-23ab, miR-26ab, miR-320, miR-324-5p, miR-329,
					miR-381, miR-539

γ1	GABRG1	3428	0	240	miR-203, miR-218, miR-379, miR-410, miR-455, miR-488

γ2	GABRG2	2106	miR-150, miR-103/107	NA	N/A

γ3	GABRG3	96	0	114	miR-15b, miR-16, miR-195, miR-26ab, miR-322, miR-497

π	GABRP	1178	0	NA	N/A

δ	GABRD	433	0	393	miR-145, miR-19ab, miR-24, miR-328, miR-365

ε	GABRE	1485	miR-22	NA	N/A

θ	GABRQ	80	0	NA	N/A

ρ1	GABRR1	464	0	NA	N/A

ρ2	GABRR2	113	0	191	0

ρ3	GABRR3	N/A	N/A	271	miR-191

The conserved microRNAs targeting GABA receptors are predicted by Targetscan 5.2 http://www.targetscan.org/ and miRanda software (http://www.microrna.org). The number in parenthesis is the number of the binding sites and none means one binding site. N/A means no information is available in TargetScan or miRanda

For β-subunits, we found two binding sites for miR-30a/30a-5p/30b/30b-5p/30/384-5p and one binding site for miR-103/107. There were 15 binding sites for 15 microRNAs on β2 and 5 binding sites for 7 microRNAs on β3. For γ-subunits, we found two binding sites on γ2 and no binding sites on γ1 and γ3, There was only one binding site for ε and no binding sites on π-, δ-, θ-, ρ1- and ρ2-subunits. In general, the "common" subunits (α, β, and γ) had more miRNA target sites than the "rare" subunits (δ, θ, ε, π, and ρ). This is probably because these subunits had shorter 3'-UTRs, in particular for ρ-subunits.

We also used another software, miRanda to predict the microRNA that target to GABA receptors (Table 3). In general, miRanda predicted more microRNAs than TargetScan. There were some common microRNAs that were predicted by both software. For example, miR-137, miR-181, miR-203, and miR-216a for α1; miR-103, miR-107, miR-30, and miR-384-5p for β1; and miR-204, miR-211, miR-23, and miR-26 for β3.

We further utilized a recently developed software, miRWalk [33], to predict the miRNAs targeting 3'-UTR and open reading frame (ORF) of GABA receptors. The results are presented in Table 4. Obviously, this method is less stringent compared to TargetScan and miRanda, since the miRWalk query yielded 79 miRNAs for GABA receptor α1 subunit in comparison with only 6 by TargetScan and 29 by miRanda.

**Table 4:** Predicted microRNAs targeting 5'-UTR, ORF and 3'-UTR region using miRWalk software

GABA receptor subunits	Entrez Gene symbol	MicroRNAs targeting 5'-UTR	MicroRNAs targeting ORF	Numbers of microRNA targeting 3'-UTR with *p*-value < 0.05
α1	GABRA1	miR-326, miR-28*, miR-29b-1*,	miR-341, miR-503, miR-150, miR-378	79
		miR-539, miR-542-5p, miR-147,		
		miR-423, miR-598-5p		

α2	GABRA2	N/A	N/A	N/A

α3	GABRA3	miR-27b, miR-27a, miR-185,	miR-350, miR-431, miR-542-3p, miR-322, miR-323*,	0
		miR-343, miR-346, miR-17-5p,	miR-140, miR-148b-3p, miR-29a*, miR-152, miR-497	
		miR-93, miR-128, miR-143,		
		miR-291a-5p, miR-20b-5p		

α4	GABRA4	N/A	N/A	N/A

α5	GABRA5	miR-345, miR-22, miR-451,	miR-24-1*, miR-24-2*, let-7d, miR-346, miR-153,	37
		miR-541, miR-369	miR-296, miR-376c, miR-466c	

α6	GABRA6	0	miR-126*, miR-743b, miR-323*, miR-330*, miR-21*,	0
			miR-153, miR-880	

β1	GABRB1	miR-323, miR-219-2	miR-140, miR-351, miR-324-5p, miR-325-3p, miR-7a*,	26
			miR-10a-5p, miR-125a-5p, miR-125b-5p, miR-376b-5p,	
			miR-384-3p	

β2	GABRB2	0	miR-20a*, miR-150, miR-297, miR-541	0

β3	GABRB3	miR-188	miR-300-5p, miR-350, miR-433, miR-881, miR-672,	17

γ1	GABRG1	miR-497, miR-322, miR-103-2,	miR-182, miR-216a, miR-483, miR-327, miR-338,	miR-126*, miR-
		miR-103-1, miR-107	miR-205, miR-296, miR-320, miR-880	379, miR-742,
				miR-871

γ2	GABRG2	0	miR-182, miR-483, miR-382, miR-505	0

γ3	GABRG3	miR-151*, miR-125a-3p	miR-142-3p, miR-298, miR-15b, miR-16, miR-28,	14
			miR-34a, miR-195, miR-214, miR-290, miR-449a,	
			miR-880, miR-708	

π	GABRP	miR-28, miR-708	miR-345-5p, miR-199a-3p, miR-873	0

δ	GABRD	miR-210	miR-322, miR-338, miR-193, miR-370, miR-497, miR-873	29

ε	GABRE	0	miR-485, miR-484, miR-342-3p, miR-344-5p, miR-223,	0
			miR-671, miR-322, miR-24, miR-139-3p, miR-199a-5p,	
			miR-298, miR-483, miR-497, miR-743b, miR-672	

θ	GABRQ	0	miR-350, miR-34c, miR-92a, miR-92a, miR-300-5p,	0
			miR-92b, miR-7a*, miR-32	

ρ1	GABRR1	miR-29b-1*, miR-26c	miR-338, let-7d, miR-204*, miR-421, miR-672,	92
			miR-674-3p	

ρ2	GABRR2	miR-873, miR-134, miR-210,	miR-218*, let-7d, miR-34a, miR-204*, miR-421,	miR-191,
		miR-207, miR-380,	miR-449a, miR-431, miR-381, miR-674-3p	miR-872*

ρ3	GABRR3	miR-350, miR-30c-1*, miR-30c-	miR-338, miR-341, miR-23a*, miR-143, miR-384-5p,	miR-191, miR-
		2*, miR-148b-3p, miR-152,	miR-324-3p, miR-30c, miR-30e, miR-30b-5p, miR-30d,	344-1, miR-217,
		miR-872*, miR-874, miR-672	miR-30a, miR-204*, miR-539, miR-742, miR-873	miR-883, miR-484

MicroRNAs which target 5'-UTR, open reading frame (ORF) and 3'-UTR of GABA receptors were predicted by miRWalk software http://www.ma.uni-heidelberg.de. N/A means that no information is available in miRWalk

**Figure 1:**
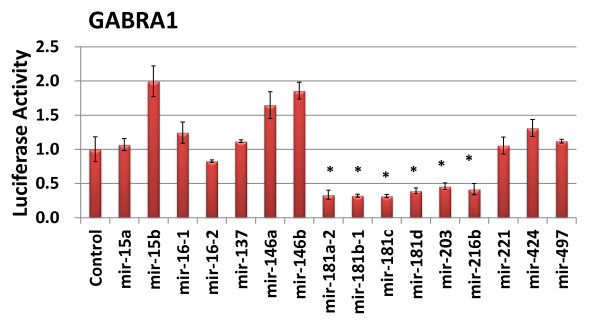
**Effect of the predicted microRNAs on the 3'-UTR reporter activity of GABA_A _receptor α1-subunit**. HEK 293T cells were transfected with the reporter and microRNA expression vectors and dual luciferase activities were assayed. The results were expressed as a ratio to the control microRNA vector. Data shown are means ± S.D. *P < 0.05 v.s. control. n = 3. Student *t*-Test.

**Figure 2:**
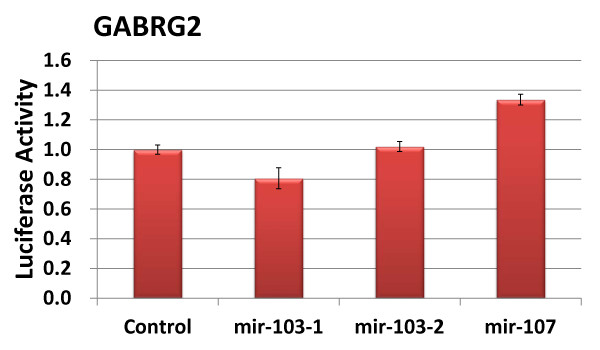
**Effect of the predicted microRNAs on the binding site reporter activity of GABA_A _receptor γ2-subunit**. HEK 293T cells were transfected with the reporter and microRNA expression vectors and dual luciferase activities were assayed. The results were expressed as a ratio to the control microRNA vector. Data shown are means ± S.D. n = 3.

We selected two subunits, α1 and γ2 for experimental verification of the predictions. For α1-subunit, we constructed a 3'-UTR reporter vector, in which the 3'-UTR of α1-subunit was placed after a *Renilla *luciferase reporter gene (Table 2). For γ2-subunit, we cloned the predicted binding site of miR-103/107 into the downstream of a *Renilla *luciferase reporter gene. We then co-transfected a microRNA expression vector with the reporter into HEK293T cells to see whether the microRNA depressed the reporter activity. The firefly luciferase pGL3 vector was used for normalization. As shown in Figure 1, among the predicted microRNAs tested, miR-181, miR-216, and miR-203 inhibited the reporter activity. Four miR-181 isoforms, a-2, b-1, c and d-1 generated the same mature miR-181 and all of them depressed the reporter activity. All other microRNAs tested had no effects. The results suggest that miR-181, miR-216, and miR-203 are the micoRNAs that regulates GABA receptor α1-subunit. It is noted that miR-15, miR-16, miR-146, miR-221, miR-424 and miR-497 were predicated to target the GABA receptor α1-subunit by an earlier version (v.4.2) of TargetScan. Some of the miRNAs such as miR-181 are expressed in astrocytes naturally expressing GABA receptors [34]. For γ2-subunit, we did not find major effects of miR-103-1, miR-103-2, and miR-107 on the reporter activity (Figure 2).

It should be noted that we did not measure miRNA levels in the miRNA-overexpressed cells. Thus, there are possibilities that some of miRNAs may not be over-expressed in the experimental set-up; particularly for these miRNAs that had no effect on 3'-UTR reporter activity. However, the transfection efficiency is 90-100% under our experimental conditions based on the GFP reporter expression encoded in the same miRNA expression vector. Additionally, the effect of a miRNA on the luciferase activity does not necessarily mean that it was a direct effect on the binding of a miRNA to the 3'-UTR reporter construct. A miRNA could have indirect effects. The mutations of seed sequences in the miRNA binding sites are needed to exclude indirect effects. Further studies are also needed to see whether the overexpression of miR-181, miR-203, and miR-216 in a physiologically relevant cell type modifies GABA receptor expression, and whether these miRNAs are differentially regulated in diseased states.

It is also interesting to note that miR-15b and miR-146a/b actually increased the 3'-UTR reporter activity. It has been reported that miRNA increases translation [35]. However, it is also possible that this is a result of indirect effects.

We have previously shown that the activation of GABA receptors promotes fetal lung development [13]. The inhibition of miRNAs that target GABA receptors may increase receptor density and thus sensitivity of GABA receptors, which may benefit the development of therapy in treating diseases related to developmental anomalies.

## Conclusions

In summary, computational approaches predict many microRNA binding sites on the 3'-UTR of GABA_A _receptors, but not on these of GABA_C _receptors. 3'-UTR reporter assays only verified miR-181, miR-216, and miR-203 as the microRNAs that target GABA receptor α1-subunit among 10 microRNAs tested. These studies reinforce that micoRNA target prediction needs to be verified experimentally. The identification of microRNAs that target to GABA receptors provides a basis for further studies of post-transcriptional regulation of GABA receptors.

## Competing interests

The authors declare that they have no competing interests.

## Authors' contributions

CZ, TW and HM carried out experiments. CZ, CH and XX analyzed data and performed target predictions. LL conceived of the study, and participated in its design and coordination. LL and CZ drafted the manuscript. All authors read and approved the final manuscript.
